# *search GenBank*: interactive orchestration and *ad-hoc* choreography of Web services in the exploration of the biomedical resources of the National Center For Biotechnology Information

**DOI:** 10.1186/1471-2105-14-73

**Published:** 2013-03-01

**Authors:** Dariusz Mrozek, Bożena Małysiak-Mrozek, Artur Siążnik

**Affiliations:** 1 Institute of Informatics, Silesian University of Technology, Akademicka 16, Gliwice, 44-100, Poland; 2 Biotechnology Center, Silesian University of Technology, Akademicka 2A, Gliwice, 44-100, Poland; 3 IBM Competence Center, Silesian University of Technology, Akademicka 16, Gliwice, 44-100, Poland

**Keywords:** NCBI entrez, Entrez databases, Entrez search engine, Entrez programming utilities, Data exploration, Data searching, Data querying, Web services, Orchestration, Choreography

## Abstract

**Background:**

Due to the growing number of biomedical entries in data repositories of the National Center for Biotechnology Information (NCBI), it is difficult to collect, manage and process all of these entries in one place by third-party software developers without significant investment in hardware and software infrastructure, its maintenance and administration. Web services allow development of software applications that integrate in one place the functionality and processing logic of distributed software components, without integrating the components themselves and without integrating the resources to which they have access. This is achieved by appropriate orchestration or choreography of available Web services and their shared functions. After the successful application of Web services in the business sector, this technology can now be used to build composite software tools that are oriented towards biomedical data processing.

**Results:**

We have developed a new tool for efficient and dynamic data exploration in GenBank and other NCBI databases. A dedicated *search GenBank* system makes use of NCBI Web services and a package of Entrez Programming Utilities (eUtils) in order to provide extended searching capabilities in NCBI data repositories. In *search GenBank* users can use one of the three exploration paths: simple data searching based on the specified user’s query, advanced data searching based on the specified user’s query, and advanced data exploration with the use of macros. *search GenBank* orchestrates calls of particular tools available through the NCBI Web service providing requested functionality, while users interactively browse selected records in *search GenBank* and traverse between NCBI databases using available links. On the other hand, by building macros in the advanced data exploration mode, users create choreographies of eUtils calls, which can lead to the automatic discovery of related data in the specified databases.

**Conclusions:**

*search GenBank* extends standard capabilities of the NCBI Entrez search engine in querying biomedical databases. The possibility of creating and saving macros in the *search GenBank* is a unique feature and has a great potential. The potential will further grow in the future with the increasing density of networks of relationships between data stored in particular databases. *search GenBank* is available for public use at http://sgb.biotools.pl/.

## Background

### Introduction

Not so long ago, the cooperation of medical science and informatics was not so common, but nowadays, collaboration of scientists from completely different fields of science is not unusual. Moreover, the cooperation of scientists from different domains initiated the formation of intermediate and interdisciplinary scientific fields; those which can combine knowledge and experience from apparently distant research areas. It appears that without the cooperation across boundaries resulting from differences between the various fields of science, the current image of scientific work would look quite different.

Several years ago, medicine and life sciences began to generate a huge amount of data. This unimaginably large amount of data had to be processed and stored in some way. Various research centers around the world began to create data repositories trying to solve the problem of collecting and processing large volumes of biological information. The solutions and techniques, which focus on storing medical data, are increasingly fine-tuned, due to the fact that the amount of information is constantly growing. One of the major reasons for the creation of biological databases is the very large amount of genetic information, including nucleotide sequences. Since 1981, when the Sanger sequencing method was invented, the problem of storing genetic information has been prevalent. GenBank [1,2] is one of the most famous databases in the world that stores genetic information. GenBank and also other biomedical databases are maintained by the National Center for Biotechnology Information (NCBI, http://www.ncbi.nlm.nih.gov). The NCBI not only hosts the databases, but also provides a uniform system for data retrieval and additional programming tools that allow software developers to create specialized applications for biomedical data integration.

### Retrieving information from NCBI databases

The most commonly used system for searching and retrieving information from databases that are maintained by the NCBI is Entrez [3,4]; the Entrez system is also a tool for indexing records in NCBI databases. The first version of the system was distributed on CD-ROM (1991). At that time, Entrez provided data on nucleotide sequences from the GenBank, amino acid sequences from the Protein database [5], which stored protein sequences corresponding to nucleotide sequences in the GenBank, and also scientific abstracts from the PubMed database [6,7].

**Figure 1:**
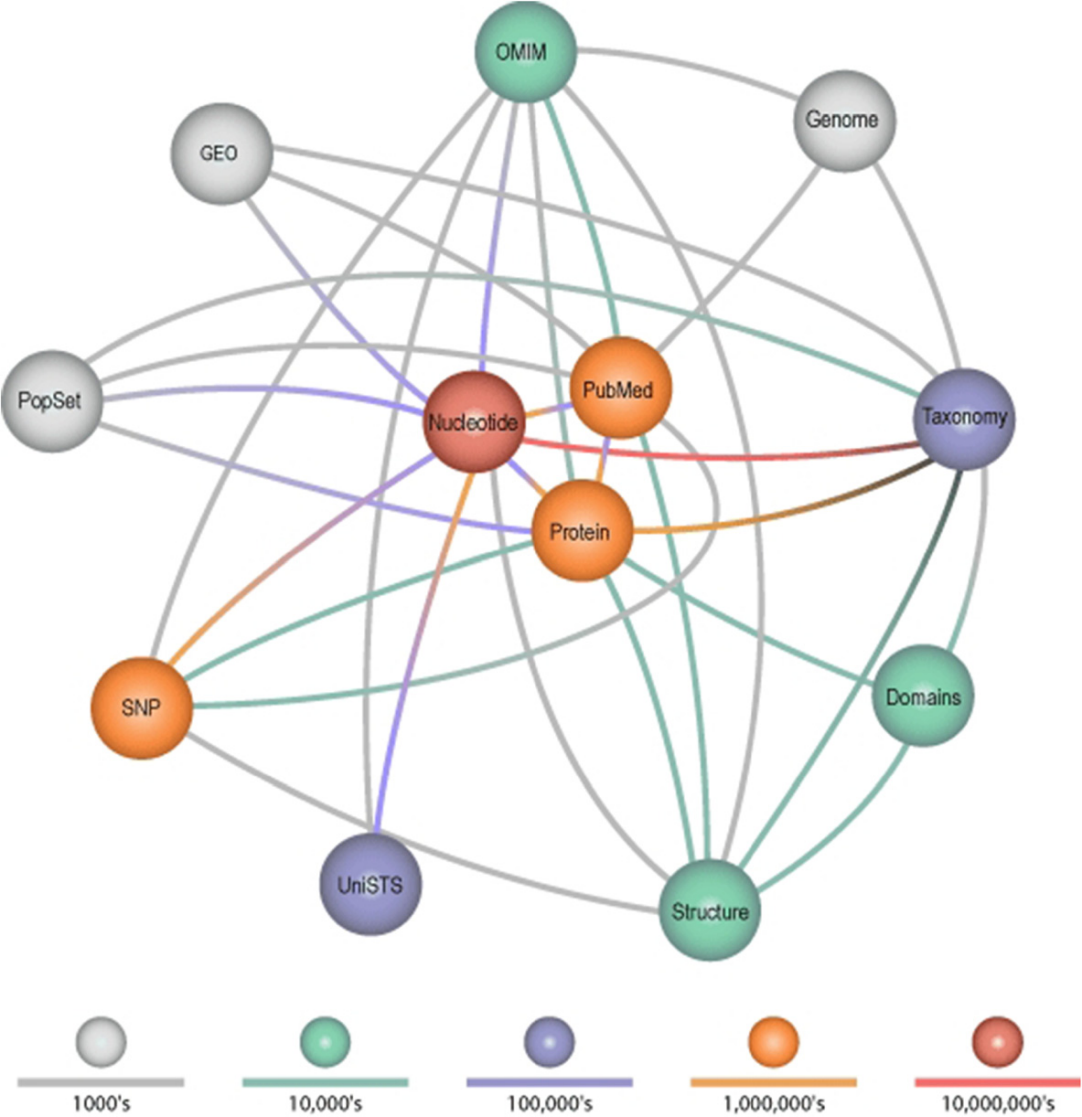
**Relationships between database nodes in NCBI Entrez.** Each node represents an Entrez database. Entrez databases group data objects of the same type, e.g. published articles in PubMed or nucleotide sequences of DNA/RNA in the Nucleotide database. Links between nodes reflect possible relationships between data, e.g. given an annotated nucleotide sequence we can quickly find the protein that is the product of its translation, and how we can traverse from one database to another discovering more about the particular object. Figure reproduced from [Ostell J., 2002].

Functioning of the Entrez system is based on the connections between nodes that correspond to specific databases. These nodes and the structure of connections between them in the Entrez system are presented in Figure 1.

The system is constantly being developed and improved; the number of nodes supported by the Entrez is increasing all the time. The original system, which contained three nodes, i.e. GenBank (Nucleotide), Proteins and PubMed, has evolved in recent years, adding new nodes, including [4]:

• Taxonomy [8], organized around the names and phylogenetic relationships between organisms;

• Structure [9], organized around the three-dimensional structures of proteins and nucleic acids;

• Genome [10], representing completely sequenced organisms and those for which sequencing is in progress, together with links to genomic data available for these organisms;

• PopSet [5], a set of DNA sequences that have been collected to analyze the evolutionary relatedness of a population;

• OMIM [11], which is a database of all known diseases with a genetic basis;

• SNP (dbSNP) [12], the Single Nucleotide Polymorphism database – a public-domain archive for a broad collection of simple genetic polymorphisms.

Access to these resources can be achieved by a graphical user interface of the NCBI Entrez system or by using NCBI Web services.

### Web services

A Web service is a software system designed to support interoperable machine-to-machine interactions over a network [13]. Web services provide a way in which we can build distributed web-accessible programs that are key components for the implementation of different types of software applications in the Service-Oriented Architecture (SOA) [14]. Unlike traditional client/server applications, Web services do not provide the Graphical User Interface (GUI). They provide their programmatic interfaces across the network in order to share processes, data and operational logic. Descriptions of these interfaces are published by the Web services creators and can be discovered and then used by Web services consumers, i.e. software developers. Software application developers can enrich their programs by using functionality that is available through the particular Web service.

Web services are widely used in business software applications [15]. What decides on their popularity is the fact that they provide the interoperability between different software applications, running on a variety of platforms and/or frameworks [13]. This is achieved by the use of several technologies, including: XML (Extensible Markup Language), which allows a structure to be described and assigned to the transferred data [16], WSDL (Web Services Description Language), which is used to describe the functionality offered by a Web service [17], SOAP (Simple Object Access Protocol), which allows the exchange of messages and data between client applications and Web services and also to call operations shared by the Web service [18], and UDDI (Universal Description, Discovery and Integration), which is a mechanism to register and locate web service applications [19].

### Web service orchestration and choreography

The availability and popularity of Web services supported by the development of associated standards, protocols and technologies, like XML, SOAP, WSDL and UDDI, provided the opportunity for building complex processes that are based on the message flow between many distributed Web services. However, the construction of such systems requires the appropriate coordination of all components taking part in the composite process. Orchestration and choreography permit this task. Orchestration [14,20] refers to a process of coordination regarding how component Web services will be invoked, what information they will receive and what information they will return. The interaction between the coordinating service and Web services is carried out using messages. A central middleware service, called an *orchestration engine* or *orchestrator*, coordinates the flow of messages during the process and models various communication paths. It maintains complete control over the running process and mediates the exchange of data. Choreography [20] is similar to orchestration, but the main difference is that there is no central coordination between calls of particular Web services. Choreography is not described from the view of the single participant (orchestrator), but from the global perspective of the cooperating participants [21]. The use of a particular coordination method depends on the situation and the complex process that is modeled.

There are several tools that allow the development of complex workflows and the coordination of information flow between Web services, including Taverna [22], SADI [23], Altova MapForce [24], and others. Taverna is a universal, open-source tool for designing and running scientific workflows. These workflows combine different Web services, local services and scripts that can be used to access various biological databases and to perform different analyses in molecular biology and bioinformatics. SADI is a framework for the discovery of, and interoperability between, distributed biological data and other bio-oriented analytical resources [23]. SADI adds semantics to descriptions of interfaces of existing Web services in order to simplify and automate service design and deployment. Altova MapForce is a domain-independent, visual data mapping tool for advanced data integration projects. It allows the user to design the flow of data between various data sources, transform the data and generate the code of the transformation. Web services can be one of the data sources and MapForce allows the construction of complex pipelines combining these Web services. MapForce is a commercial tool. Microsoft SQL Server Integration Services (SSIS) [25] is another commercial product allowing the integration and transformation of data from various data sources, including Web services. Workflows similar to those built in Taverna may contain many component tasks accessing Web services that can be invoked in the planned sequence.

In this article, we present a new tool for the efficient and dynamic exploration of data in GenBank and its allied databases. We have designed and developed a special *search GenBank* system and web portal, which enables searching for information in GenBank and also in other NCBI databases through the interactive orchestration of NCBI Web services. Moreover, by using macros, users transparently design their own choreography of the Web service utilities, extending the possibilities of standard data searching of the NCBI Entrez.

## Implementation

While exploring biomedical databases in the face of the growing number of bio-resources available worldwide, we have to answer one fundamental question: whether to integrate the available data or use existing systems through the shared endpoints? Since the integration of biological data resources and tools requires investments in computer infrastructure and its maintenance, in our solution we adopted the latter approach. *search GenBank* uses Web services and a set of available utilities in order to explore biomedical databases of the NCBI.

*search GenBank* provides three paths of data exploration:

- simple searching based on the specified user’s query,

- advanced searching based on the specified user’s query, and

- advanced exploration with the use of macros.

Each of the above mentioned paths starts with the initial query, which is specified by a user. This query can be given as a single word or phrase, which usually takes place in the simple searching mode, or can have dedicated syntax specified by NCBI, which is typically used in the advanced searching mode. Advanced exploration with the use of macros allows not only the submission of a query to one particular database, but also traversing a predetermined path between various databases and discovering related information. From a technical point of view, we can treat simple and advanced searching modes as a special case of the advanced exploration with the use of macros, wherein we use a single database for the exploration.

Query syntax and construction, how phrases provided by users are automatically broken into terms and how they are mapped to appropriate fields that are searched by the NCBI search engine are described in the following subsections. These are internal features of the NCBI Entrez search engine. However, we describe them here because they are implicitly used while invoking NCBI Web services by *search GenBank*. We also present the architecture of the *search GenBank* system and the flow of information in various paths of data exploration. Before we go deeper into functional details of the system, we will consider the following scenario.

### Motivating use case

This scenario shows one of possible exploration paths when diagnosing diseases based on the analysis of a patient’s DNA. Changes in the DNA sequence may be spontaneous or caused by chemical or physical factors. When they occur at a frequency of less than 1% of the population they are identified as a mutational change – genomic mutations. These changes in the nucleotide sequence can cause changes in the protein sequence, structure, and function, such as loss of function, or less frequently, the acquisition of a new function. Unfortunately, while recognizing the change in the DNA sequence, we can rarely clearly answer the question of how it affects the function of the protein. If a change in the DNA causes the emergence of a stop codon that ends the translation process, we can clearly state that there is a change of the protein function due to the truncated amino acid chain produced or complete loss of function if the cell degrades truncated protein. Any other change in the DNA can affect the structure and function of the protein, but the identification of the effect of the nucleotide change is primarily based on the evaluation of the patient’s phenotype or evaluation of how the presence of a disease correlates with the presence of mutation in members of the studied family. Such an analysis is typical when diagnosing many diseases which have their origin in DNA mutations.

**Figure 2:**
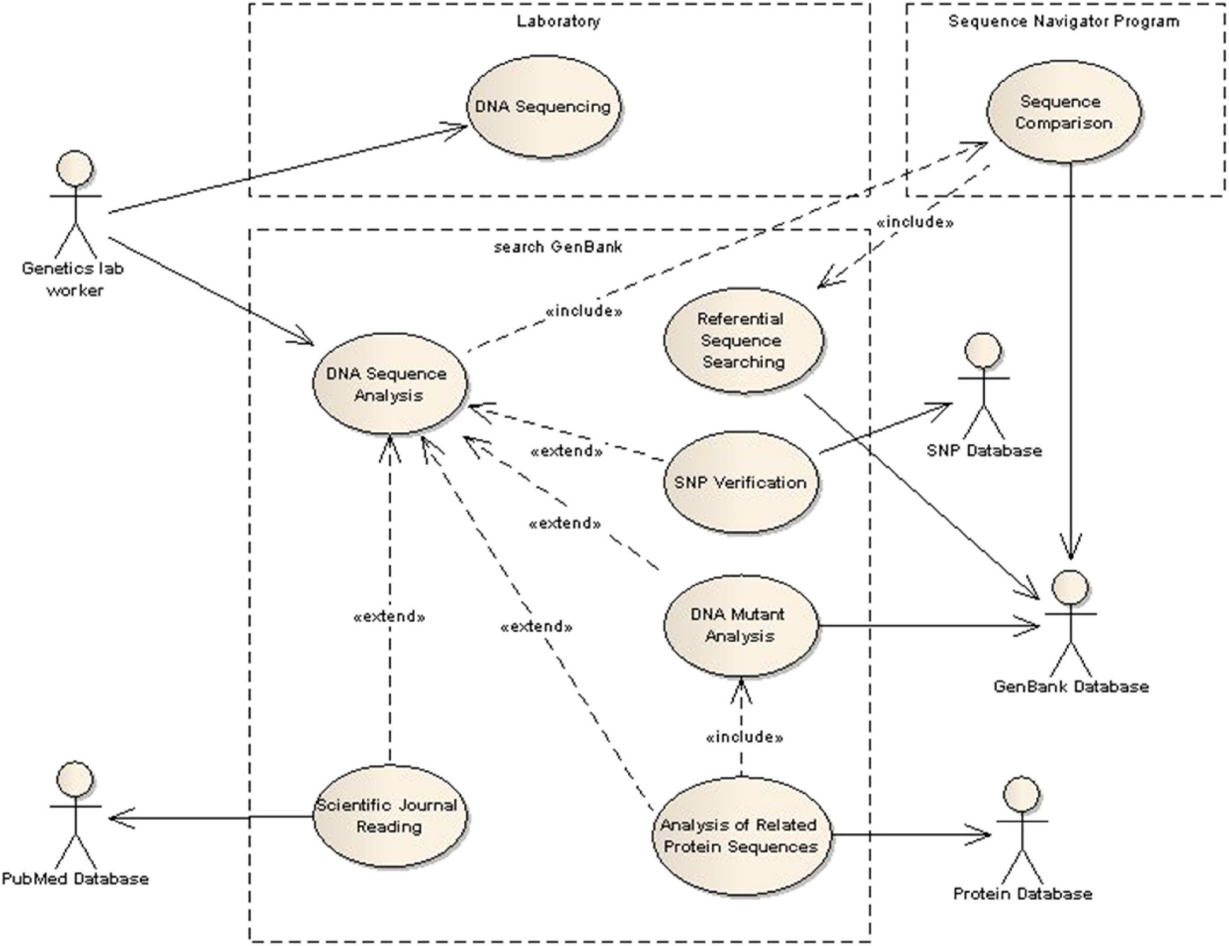
**Use case diagram describing possible data exploration while diagnosing diabetes mellitus based on the sequence of the *HNF1A *gene.** Genetics lab worker obtains the nucleotide sequence of the *HNF1A* (hepatocyte nuclear factor *HNF-1alpha*) gene in the laboratory. He/she performs the sequence analysis, which may include the comparison of the obtained gene sequence to the reference sequences from the GenBank. The sequence comparison can be done by any program, e.g. *Sequence Navigator*, and must be preceded by a referential sequence search, which can be done using *search GenBank*. If there are no DNA changes in the patient’s sequence the lab worker reports no changes. Otherwise, he/she verifies whether there are any entries in the SNP database reporting the change in such position of the gene sequence and whether the change has been described and is pathogenic (polymorphic site or mutant). In case of mutants, he/she can analyze mutant sequences by exploring records from the GenBank, related protein sequences from the Protein database and related protein 3D structures from the MMDB. At any moment, he/she may want to broaden his/her knowledge about the change by finding and reading publications from the PubMed database.

For example, mutations in the hepatocyte nuclear factor (HNF)-1alpha gene (*HNF1A*) are the most frequent cause of MODY (Maturity Onset Diabetes of the Young) [26]. When examining patients suspected of having diabetes, a diagnostician, biologist or medical doctor verifies whether there are changes in the *HNF1A* gene, and if so, what the type of change is – whether it is a mutation or a polymorphism, and whether the change has been described and is known to be pathogenic. In addition, when this change has not been previously described in a database, how it affects the protein sequence and structure, and whether there are any publications in this field that must be considered. Such analysis requires repeated access to a number of biological databases and searching links between different types of data. This example is illustrated in Figure 2.

The analysis presented in this scenario may generate various, shorter or longer exploration paths depending on the conclusions drawn from the investigation. *search GenBank* provides various exploration modes for such scenarios, and each of them begins with an initial query to particular database.

### Query syntax and construction

While searching the NCBI databases, users usually input key words or search phrases to the search box and submit them to the NCBI Entrez search engine. The strings entered into Entrez are converted to queries with the following format [27]:

Term1[field1]**Op**Term2[field2]**Op**Term3[field3]**Op**…

where: *Term*, specifies the query phrase, *field* is a searched field (always enclosed in square brackets), and *Op* is one of the available logical operators: AND, OR or NOT (operators must be written in capital letters).

Example: breast cancer **AND** human[organism]

**Figure 3:**
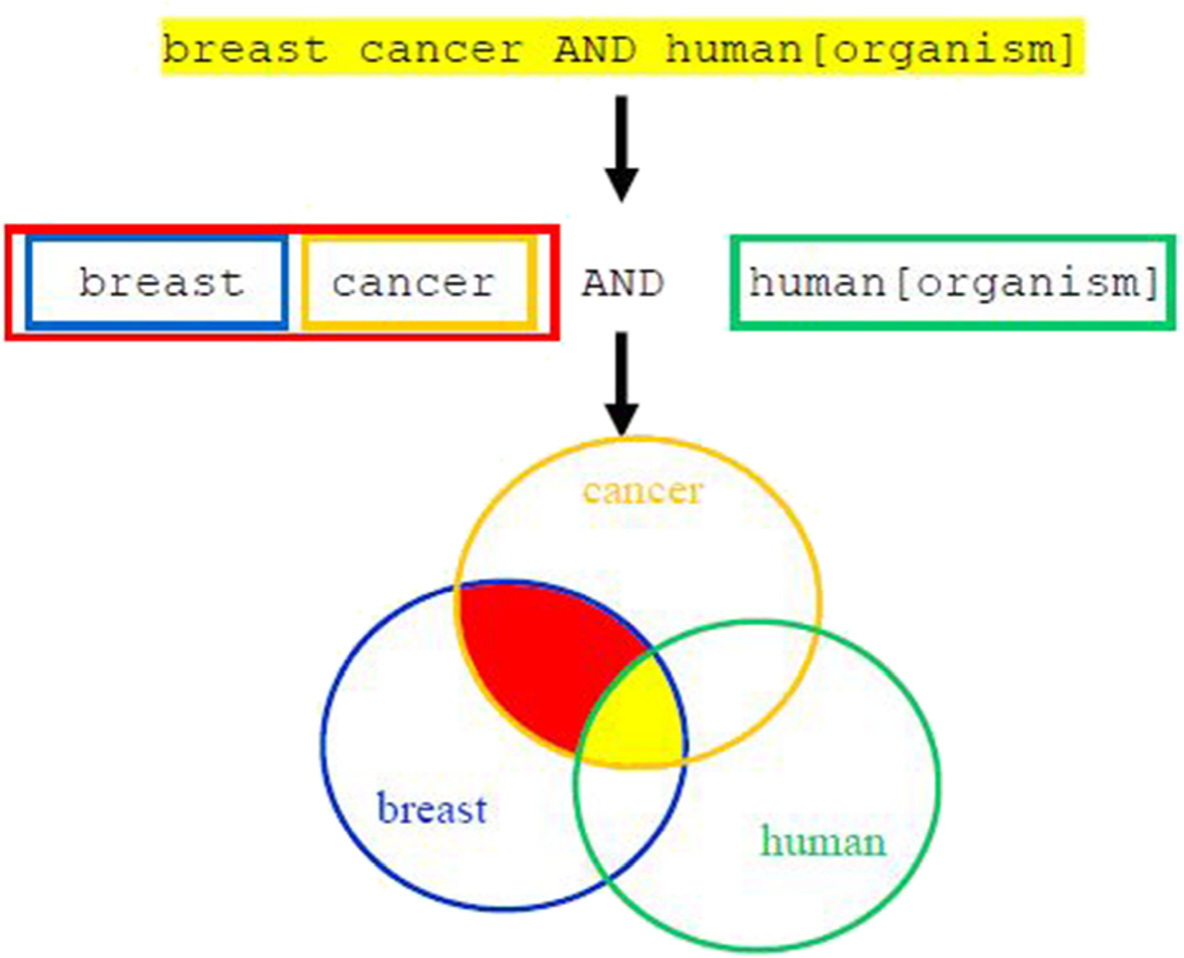
**Graphical representation of information searching in Entrez.** A given query is divided into a series of elements: first, with respect to the logical operators, and then with respect to the space symbol. In the presented example we obtain three elements: *breast*, *cancer* and *human[Organism]*. Each element of the query is processed separately generating its result set. Results are merged according to the operators used in the query – first *breast* and *cancer*, and then the common part of their result sets is merged with the result set of *human[Organism]*.

Entrez divides the query into a series of elements, which were separated in the original query by a space. If the query includes logical operators, the system will divide the query into a series of elements, first, with respect to the logical operators, and then with respect to the space symbol [27]. Each element of the query is processed separately, and search results are then merged according to the operators used in the query (Figure 3). The default logical operator is AND.

In the situation where the query consists only of a list of UIDs (unique identifiers of records in NCBI databases) or accession numbers, Entrez will return only those records to which given identifiers refer. Therefore, no additional query processing will occur.

### Automatic mapping of phrases in queries submitted to Entrez

While processing queries, the Entrez system automatically searches the database for each phrase of the query taking into account the following criteria:

1. Taxonomic node – each phrase is limited to the [organism] field or [All Fields]. For example, for the *mouse* phrase, the system automatically maps the phrase to: “Mus musculus”[organism] OR mouse[All Fields].

2. Names of journals – the database is searched against the names of journals, e.g.: science → science[Journal].

3. Name of the author – the result of the query is narrowed to [Author] field. Not every phrase can be mapped to the name of the author field, because the correct name of the author can only be the word, followed by one or two letters. For example: Mrozek D → “Mrozek D”[Author].

If the system does not return results after the auto-mapping process, then the rightmost phrase of the query is removed and mapping process is repeated until the system returns results. If, despite this, system still does not return the results, all query phrases are limited to the All Fields field, and they are joined by the logical operator AND.

In Table 1 we show examples of queries before and after the automatic mapping process.

**Table 1:** Examples of automatic mapping of phrases in queries submitted to Entrez

**Original query**	**Query after automatic mapping process**
breast cancer inhibitor	(“Clin Breast Cancer”[Journal] OR “Breast Cancer”[Journal] OR (“breast”[All Fields] AND “cancer”[All Fields]) OR “breast cancer”[All Fields]) AND inhibitor[All Fields]
cancer inhibitor breast	(“Cancer”[Organism] OR cancer[All Fields]) AND inhibitor[All Fields] AND breast[All Fields]
human rab5a	(“Homo sapiens”[Organism] OR human[All Fields]) AND rab5a[All Fields]
bos polymerase gene	(“Bos”[Organism] OR bos[All Fields]) AND polymerase[All Fields] AND gene[All Fields]
bos a polymerase gene	bos a[Author] AND polymerase[All Fields] AND gene[All Fields]

### Entrez Programming Utilities

Entrez Programming Utilities (eUtils) [27-29] is a set of eight programs running on the NCBI server side. These utilities provide a stable interface to the NCBI Entrez and NCBI resources. We can use eUtils in two ways. The first way is that our application can send a properly composed URL address to the server, which makes the tools available so that it can receive the response from the tool in the XML format. The second, more elegant way, is to use Web services that guarantee the interoperability across platforms, applications, and programming languages, and rely on standardized protocols (SOAP, WSDL, and UDDI). On its website, the NCBI makes available links to the WSDL files with a description of the Web services [29] that provide the access to eUtils.

In Table 2 we present a list of all eUtils tools with a brief description of their function [27,30].

**Table 2:** Description of available eUtils tools

**Tool name**	**Description**
EInfo	Provides information about available databases or a specific database, such as: the number of indexed records for each searched field, the date of the last update, and available links to other NCBI databases.
EGQuery	Responds to the query returning the number of records that match the specified, search phrases in any Entrez database.
ESearch	Responds to the query returning a list of unique identifiers (UID) of records that match the specified query.
ESummary	For the specified list of UIDs, returns summaries of records from a particular database.
EPost	Accepts a list of UIDs and sends it to the History Server, returning the appropriate address in the form of parameters: WebEnv and query_key.
EFetch	Responds to the list of UIDs returning complete records from a specified database.
ELink	Allows traversing between databases using related UIDs. Returns the list of UIDs of records from a destination database related to the UIDs of records from the source database.
ESpell	Returns suggestions of the correct spelling for the query entered by the user.

### Interactive orchestration of eUtils

Orchestration allows the user to combine various utilities in order to create a composite exploration process. *search GenBank* is a kind of interactive orchestrator allowing various exploration paths to be modeled. It allows users not only to submit queries to particular databases and receive results; users are also able to browse related records in other databases. Working interactively with the *search GenBank*, they translate their own logic of the analysis process into the flow of information from the *search GenBank* to the NCBI Web service and opposite. Actually, it is *search GenBank* that makes the translation of user’s navigation paths to a set of commands sent and received to and from the NCBI Web services and coordinates the compound analysis process. This interactive orchestration is available in all three modes of data exploration, which will be discussed in more details in the Results section.

For example, the basic combination of the tools included in the eUtils package is [30]:

### ESearch → EFetch/ESummary

**Figure 4:**
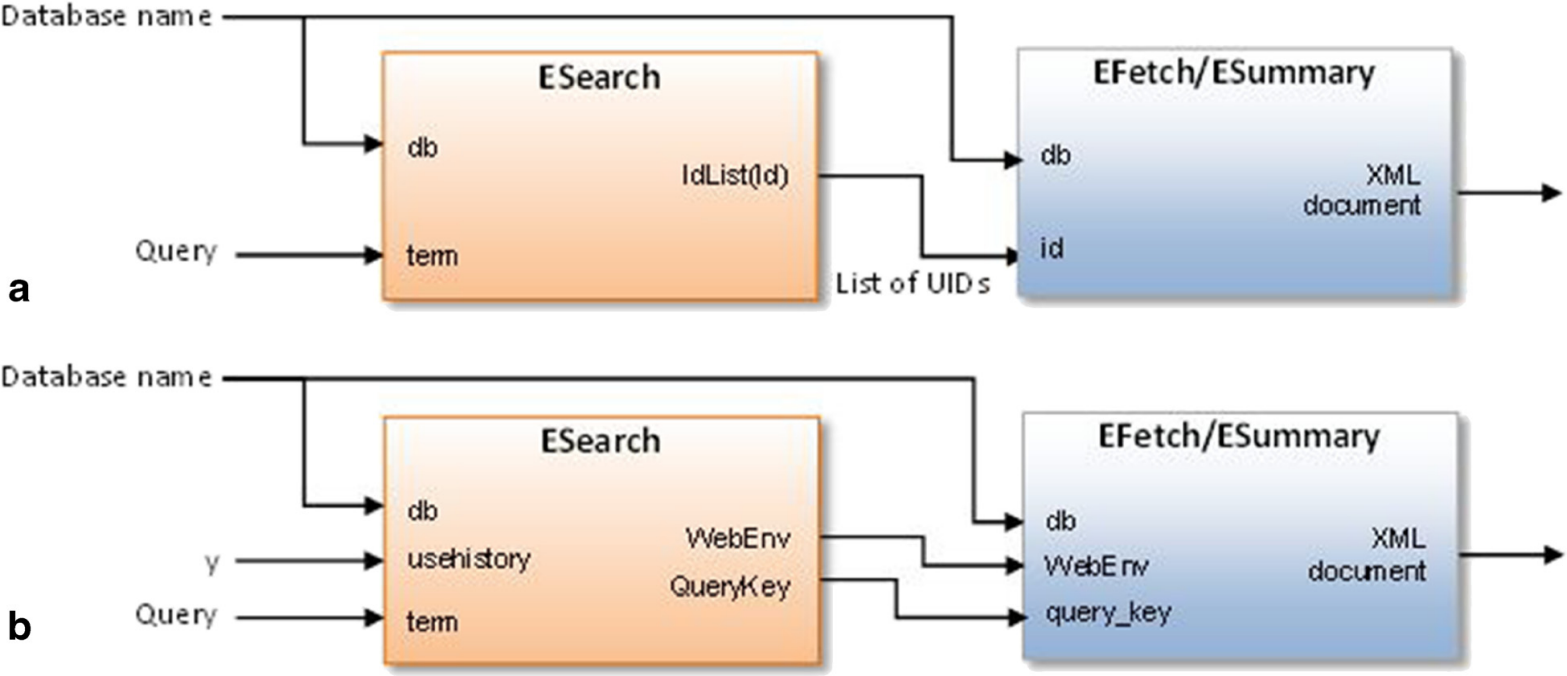
**Flow of control in the pipeline containing ESearch tool and EFetch/ESummary tool.** An initial query is executed against a chosen database by using the *ESearch* tool. The query is entered on the *term* input of the *ESearch* tool, which also requires a database name to be provided on the *db* input. If there are records in the chosen database that satisfy the query, the *ESearch* tool generates a list of record identifiers (*UID*s), which is available on the *IdList* output (**a**) or posted onto the Entrez History Server (**b**), if the *usehistory* parameter is set to *y*. Based on the list of UIDs, the *EFetch* or *ESummary* programs retrieve appropriate records from the specified database (*db* input) and return them as an *XML document*. The list of UIDs can be entered on the *id* input of the *EFetch* or *ESummary* or can be taken directly from the Entrez History Server using a pair of *WebEnv-QueryKey* parameters.

This combination allows the user to search the specified database and retrieve adequate records that satisfy the criteria contained in the given query. The ESearch program is responsible for generating a list of record identifiers (UIDs) in a given database, for those records that satisfy the given query (Figure 4). The EFetch or ESummary programs retrieve records from the specified database with identifiers according to the given list of UIDs. Calls of appropriate programs are coordinated by the *search GenBank*.

The list of UIDs can be explicitly entered on the *id* input of the next tool (Figure 4a) or can be implicitly passed to the next tool from the Entrez History Server using a pair of WebEnv-QueryKey parameters (Figure 4b). The first of these methods is very useful when users interactively choose records through the graphical user interface (GUI) and traverse between data sources passing UIDs of these records on to the next program in the pipeline. The second method allows the list of UIDs to be stored outside of the *search GenBank*, at the Entrez History Server, which is a valuable feature when there is a full navigation path, i.e. pipeline, already prepared and predetermined (see choreography in the next chapter) or when dealing with huge amounts of data. In the latter case, the use of the Entrez History Server makes it feasible to limit the number of data transferred to and from the *search GenBank* between successive invocations of Web services.

**Figure 5:**
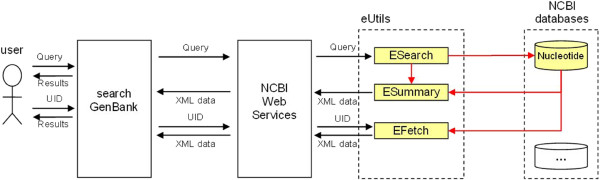
**Flow of information during data searching in the simple and advanced modes.** A user specifies a query (e.g. *hnf1a AND homo sapiens[Organism]*) and a database, against which the query will be executed (e.g. *Nucleotide*). The *search GenBank* calls the *ESearch* tool and passes the name of the database, user’s query and additional parameters as input arguments. The *ESearch* tool returns the list of identifiers for records that satisfy the user’s query. The *search GenBank* calls the *ESummary* tool, which is parameterized by messages provided by *search GenBank*, *ESearch* tool, and explored databases. The *ESummary* returns a list of records (their summaries) satisfying the user’s query. After a short review of returned records, the user decides to see full details of one of them (e.g. UID 260593720). He/she interactively triggers a call of *EFetch* tool, which retrieves data from the database for a passed record identifier *UID* and presents details of the chosen record on the *search GenBank* web page.

In Figure 5 we present the architecture of the system and sample information flow when searching in the simple or advanced searching modes. In this case, a user specifies a query as a free text or in the structured form described in previous subsections. He/she also specifies a database, against which the query will be executed (e.g. Nucleotide). The *search GenBank* calls the appropriate tool available through the NCBI Web service, the ESearch in this case, and passes the name of the database, the user’s query and any additional parameters as input arguments. The ESearch tool returns only basic information (list of UIDs) on the records that satisfy the user’s query; therefore, in order to present useful data, the *search GenBank* calls the ESummary tool, which is parameterized by messages provided by *search GenBank*, the ESearch tool, and the explored database.

Now, let us suppose that the user decides to see the full details of the presented records. In this situation, he/she interactively triggers an invocation of the EFetch tool (Figure 5) through the *search GenBank* web page. If the user wants to see related records in other database (e.g. Gene), he/she uses dedicated links, which implies an invocation of the ELink tool followed by the invocation of the ESummary tool again, passing appropriate parameters to each of the tools.

In other words, if the objective of the user is to retrieve records from one database and then find associated records in another database (or the same database), the following combination of tools must be used to accomplish this task:

### ESearch → ELink → EFetch/ESummary

In this pipeline, the ELink is responsible for generating a list of UIDs for records from a destination database, which are related to the records from the source database, against which the user’s query was executed. It is worth noting that the destination database can be the same as the source database, so that links can be followed between records of the same type, often called “neighbors”, in sequence and structure nodes [4].

Navigation paths are described in more details in the Results and Discussion section. However, we have to be aware that they involve interactive orchestration behind the scenes; that is the role of *search GenBank* as an orchestrator.

### *Ad-hoc* choreography in advanced data exploration

When the exploration path is predetermined (i.e. users exactly know how they want to traverse between data sources in order to get the desired information), the *search GenBank* system minimizes the coordination between calls of particular tools of the NCBI Web services applying choreographies. In our system, choreography defines:

- what tools will be used during data exploration,

- what resources and databases will be involved in the process,

- what is the order of using resources and tools,

- and, indirectly, what will be the message flow between components taking part in the process.

**Figure 6:**
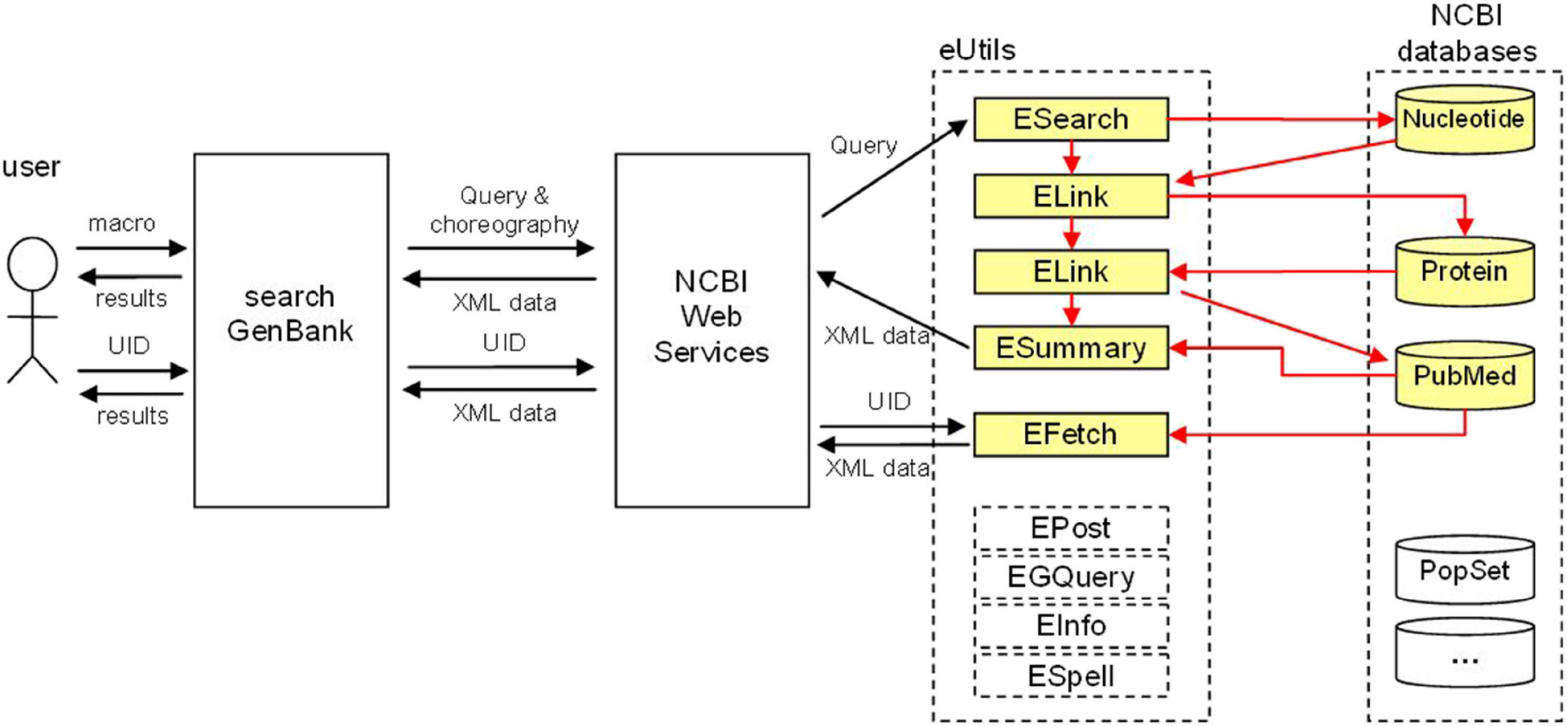
**Flow of information during advanced data exploration using macros.** A user wants to find all scientific articles related to protein sequences that are the translation products of nucleotide sequences corresponding to *HNF1-alpha* (hepatocyte nuclear factor 1-alpha). A user defines a *macro* by specifying: 1) initial *query* to be executed against the first of the chosen databases (e.g. *hnf1a[Title] AND human[Organism]*), 2) all databases that he/she wants to explore in order to find related information he/she is interested in, and 3) the order of database access (*Nucleotide*, *Protein*, *PubMed*). On the basis of the information provided by a user, *search GenBank* translates the *macro* into the *choreography* of calls of appropriate tools available through the *NCBI Web Services*. An appropriate pipeline of successive calls is created, including *ESearch*, *ELink* and *ESummary* tools. The *ESearch* component runs the *query* provided by the user against the *Nucleotide* database. Results of the query execution are passed to the first *ELink*, which searches related records in the *Protein* database, and again, the returned records (*UID*s) parameterize the call of the second *ELink* component, which finds related articles in the *PubMed* database. Finally, the summary of articles is returned by the *ESummary* component in the form of the XML document. This document is sent back to the *search GenBank*, which presents it to the user in a friendly form. After a short review of returned records, the user decides to see full details of one of them (e.g. UID 22517943). He/she interactively triggers a call of *EFetch* tool, which retrieves data from the *PubMed* database for a passed record identifier *UID* and presents details of the chosen record on the *search GenBank* web page.

In *search GenBank*, users implicitly create choreographies by designing macros. In Figure 6 we can see the architecture of the system and a sample flow of information during advanced data exploration using macros. In the example, a user defines a macro by specifying: 1) the initial query to be executed against the first of the chosen databases, 2) all databases that he/she wants to explore in order to find related information he/she is interested in, and 3) the order of database access. On the basis of the information provided by a user, *search GenBank* translates the macro into the choreography of calls of appropriate tools available through the NCBI Web services. An appropriate pipeline of successive calls is created, including ESearch, ELink and ESummary tools. In Figure 6, the ESearch component is shown running the query provided by the user against the Nucleotide database. Results of the query execution are passed to the first ELink, which searches related records in the Protein database, and again, returned records (UIDs) parameterize the call of the second ELink component, which finds related articles in the PubMed database. Finally, the summary of articles is returned by the ESummary component in the form of the XML document. This document is sent back to *search GenBank*, which presents it to the user in a friendly format.

Similarly, if we are interested in a wider spectrum of relations, for example, our goal is to find the amino acid sequences associated with genes that are, in some way, related to nucleotide sequences from the population set of sequences belonging to the mouse, then the number of calls to the ELink tool is three:

### ESearch → ELink → ELink → ELink → EFetch

For such a case, the ESearch returns a list of UIDs for all of the population sets of sequences for the mouse retrieved from the PopSet database. The first call of ELink finds a list of UIDs of nucleotide sequences from the Nucleotide database, which were included in the found population sets. The second call of ELink generates a list of genes from the Gene database, which are linked to these nucleotide sequences. The result of the last call of ELink is a list of UIDs of proteins from the Protein database that are associated with the previously obtained list of genes.

Similarly to the interactive orchestration in the simple and advanced searching modes, the destination database can be the same as the source database if we want to find neighbors in sequence and structure nodes.

## Results and discussion

*search GenBank* provides a web portal that allows users to search and retrieve information from NCBI databases. In the beginning, it was designed to display only nucleotide sequences of the GenBank database, hence the name of the portal. At that time, we cooperated with the Department of Internal Diseases, Diabetology and Nephrology, Medical University of Silesia, Zabrze, Poland, as we needed referential sequences for the comparison of DNA samples obtained from patients’ serum in order to find mutations while diagnosing different types of diabetes mellitus. However, since this time we have redeveloped the entire system and the web portal, extended its functionality towards more sophisticated searching, which includes queries and macros and the involvement of other NCBI databases. However, for historical reasons, the name of the portal remained unchanged.

The web portal has been tested for the following databases (bold names indicate databases for which we have prepared a representative form of their records):

- **Nucleotide** – the main database of nucleotide sequences (including GenBank),

- **dbEST**[31] – the database of EST sequences (Expressed Sequence Tag),

- **dbGSS**[5] – the database of GSS sequences (Genome survey sequence),

- Genome – representing completely sequenced organisms and those for which sequencing is in progress,

- **PopSet** – the database of sequences from a single population study,

- Taxonomy – the taxonomy database,

- Gene [32] – the database of known genes,

- OMIM – the database of all known diseases with genetic components,

- SNP – the database of single nucleotide polymorphisms,

- PubMed – the database of citations and abstracts for biomedical publications,

- PMC [33] – the database of free full-text biomedical and life sciences journal literature,

- Journals [5] – the database of scientific journals,

- **Protein** – the database of amino acid sequences.

The web portal allows the exploration of resources from the databases mentioned above in the same manner, as it is resolved in the NCBI Entrez service (http://www.ncbi.nlm.nih.gov/guide/). Apart from the standard search method, in which a user manually enters a query to the search box, we also made available an advanced search module, which allows the user to define appropriate limits and constraints restricting the results of the query.

The *search GenBank* internet portal is also equipped with a module for building macros. Macros are used in order to automate searching other databases for records which are related to those that are the result of the initial query. This module is an innovative part of *search GenBank* and we did not find any equivalent software that would offer the same functions.

The application interface was designed and composed taking into account the expectations of modern users of Internet services and is compatible with the accepted principles of transparency in presenting information on web sites.

The program allows registered users to utilize the simple system for saving entered queries and built macros. This amenity introduces to the service the possibility of reusing saved elements, without remembering the configuration of built macros or writing a complex query again.

The web portal is available at: http://sgb.biotools.pl. In the following sections we will present the functionality of the *search GenBank* system.

### Quick and simple data searching

**Figure 7:**
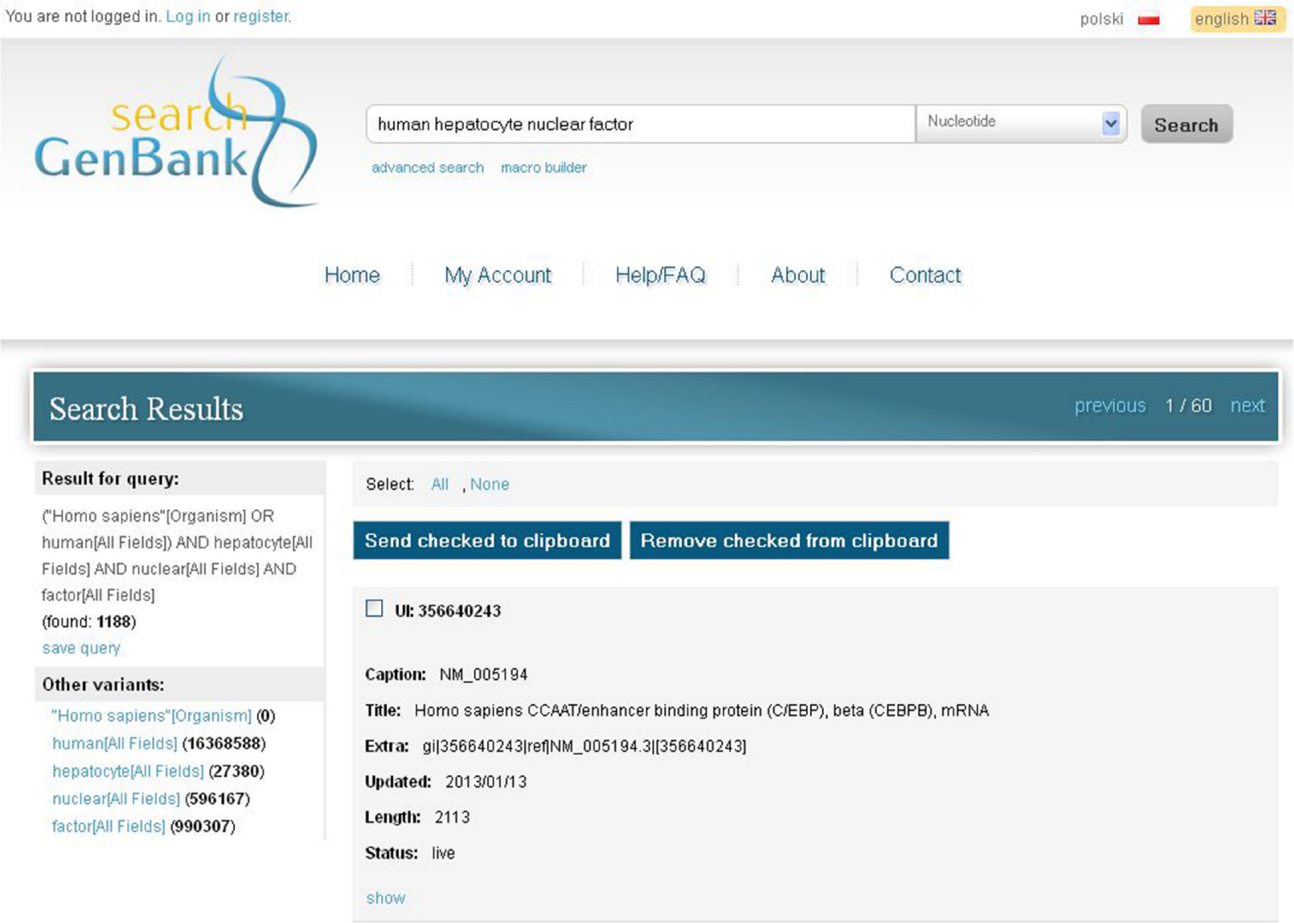
**Results of sample query in *search GenBank *service.** Results for searching genetic data in the *Nucleotide* database for the sample phrase *human hepatocyte nuclear factor.* A short summary for one of 1188 records satisfying the query is presented in the center. The sample phrase was transformed to the query presented in section *Results for query*. The section *Other variants* shows the list of suggested alternative queries together with the expected number of results. Users can show full details of a record by choosing a *show* link under the summary of the record, choose records by selecting the checkbox next to their identifiers (*UI*) and send records to the clipboard by pressing the *Send checked to clipboard* button.

Simple searching is available on each site of the *search GenBank* exploration service. There is a text field at the top of every page (see e.g. Figure 7), where users can enter a single word or a phrase that they want to find. Additionally, from the drop-down list on the right side of the query field, they can select the name of the database to which they want to submit the query. In Figure 7 we present the results for searching genetic data in the *Nucleotide* database for the sample phrase *human hepatocyte nuclear factor.*

It is worth noting the form into which the given phrase *human hepatocyte nuclear factor* has been transformed. The following query enhancement blocks may appear on the left side of the results pane:

- *Result for query* – shows the user’s query translated to the form accepted by NCBI Entrez search engine, together with the number of records satisfying the query, and gives also the possibility to save queries (*save query*);

- *Other variants* – shows the list of suggested alternative queries together with the expected number of results;

- *Did you mean?* – is optional and points to possible errors in the spelling of the provided query.

**Figure 8:**
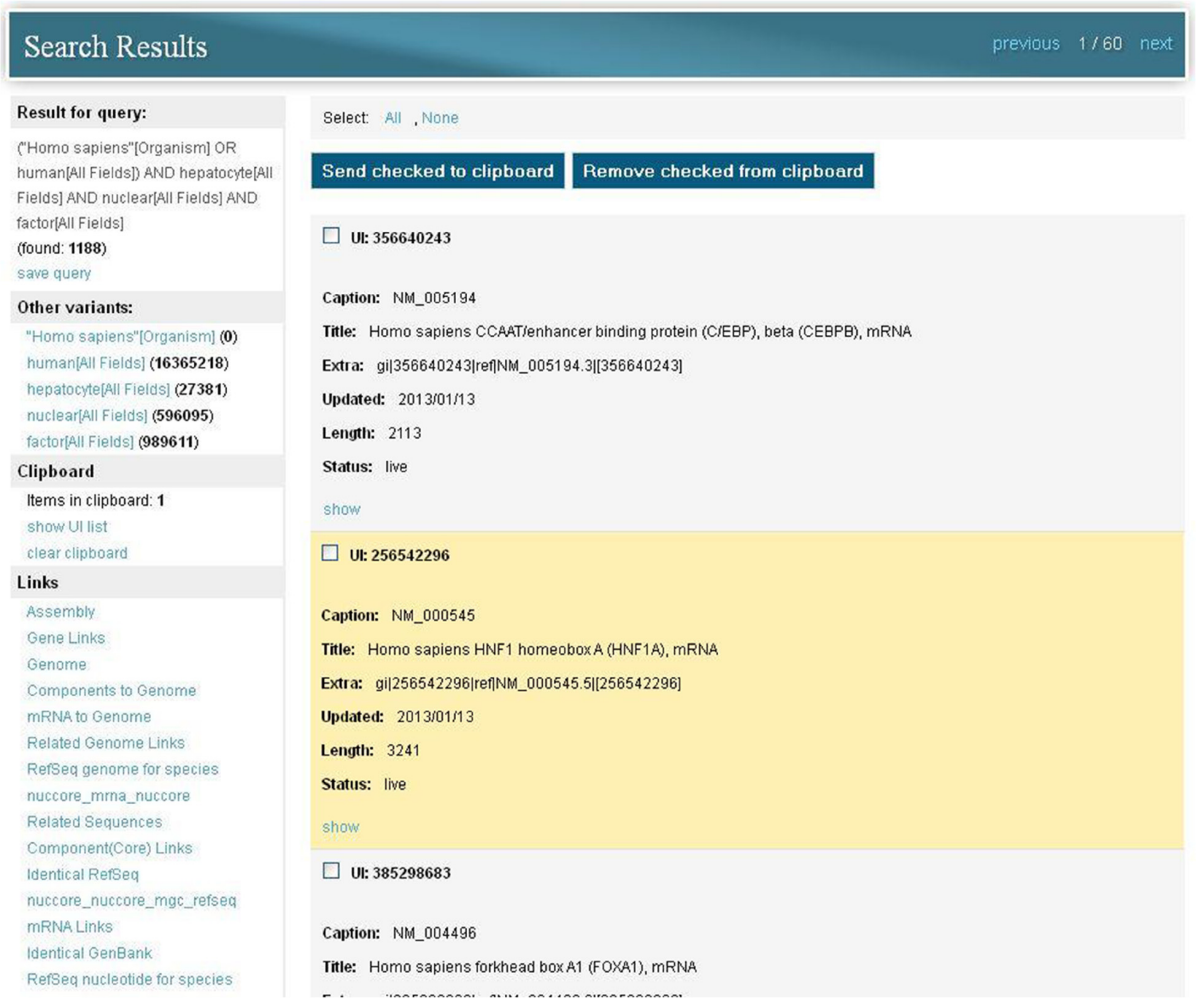
**Content of clipboard and links to other data sources available through *search GenBank.*** Records added to the clipboard are highlighted in light orange, e.g. UI: 256542296. For browsed records and records stored in the clipboard, users can check whether there are related records in other databases or even in the same database (neighbors). This is possible through a series of links available in the *Links* section on the left-hand side of the application window.

In fact, the simple search mode in *search GenBank* is similar to the standard search method used in NCBI Entrez, providing similar functionality, but organized differently on the website. Records that have been returned as a result of a query can be browsed and added to the clipboard (Figure 8, *Clipboard* section). When we browse a single record or the content of the clipboard, it is also possible to check whether there are any records in other databases that are related to the browsed record (cross-database queries). For example, if we want to search genetic data from the *Nucleotide* database, we can also link this data with protein sequences from the *Protein* database or with bibliographic data from the *PubMed* database. This is done through a series of links that are made available to the user on the left-hand side of the application window (Figure 8, *Links* section).

It is also possible to find neighbors (related records of the same type) by using available links. For example, browsing a genetic record from the Nucleotide database we can find highly similar sequences (by BLAST score) to current records or genomic sequence records that have the current mRNA record as an annotated feature marking the exons of genes.

### Advanced data searching

Terms extracted from the phrases entered by a user are not always mapped to the database fields, which the user would expect (see Figure 7, *Results for query* section). As a result, the list of returned records can often be too large or may contain inappropriate records. Advanced searching in the *search GenBank* portal allows for the precise composition of queries, using search fields that are specific for the selected database and additional limiting elements. Queries are constructed according to the rules presented in the section *Query syntax and construction*, typically combining many simple filtering criteria by the use of Boolean operators. For example:

“hnf1a”[GENE] AND “human”[ORGN] AND “MODY”[ALL]

**Figure 9:**
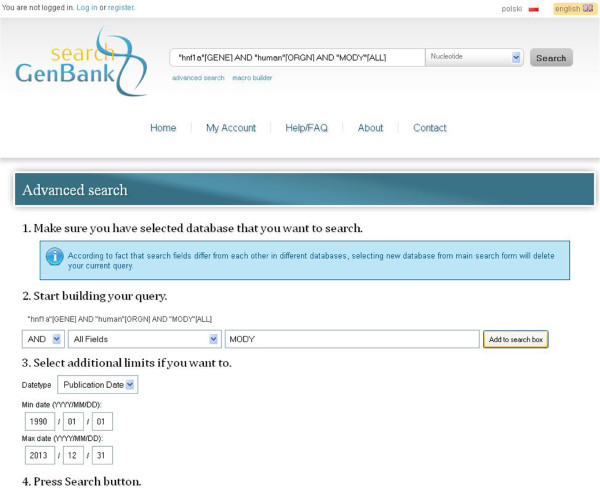
**Query builder in advanced searching mode in the *search GenBank *service.** Users can build complex queries using the query builder. Query builder allows composing a query by adding simple filtering phrases or terms that are joined by logical operators. Users are able to choose the logical operator and a database field that will be searched to find the given phrase or term (point 2). They can also set additional limits on the records that should be returned in the result set (point 3). In the presented example, a user wants to find all nucleotide sequences in the *Nucleotide* database that correspond to *HNF1A* gene in *human* organism and are involved in the maturity onset diabetes of the young (*MODY*). He/she intends to search the term *hnf1a* in the *Gene Name* field (*GENE*), term *human* in the *Organism* field (*ORGN*), and term *MODY* in *All fields* (*ALL*). All filtering conditions are joined by the *AND* operator. Composed query, i.e. *“hnf1a”[GENE] AND “human”[ORGN] AND “MODY”[ALL]*, is visible in the query/search box next to the database drop-down list at the top of the page. Additional constraints are set on the *Publication Date* in point 3.

In this example, a user wants to find all nucleotide sequences in the *Nucleotide* database that correspond to *HNF1A* gene in *human* organism and are involved in the maturity onset diabetes of the young (*MODY*). The *search GenBank* portal provides an appropriate query builder, which allows the construction of complex queries. The query builder is presented in Figure 9.

Briefly, the advanced searching process can be completed in the following steps:

1. Choose the database from the main query form (database drop-down list at the top of the page).

2. Create your own complex query:

a. Choose a logical operator combining appropriate phrases of the query.

b. Choose the search field.

c. Enter the search phrase.

d. Click *Add to search box* button.

e. Repeat steps **a** through **d**, if necessary.

3. Press the *Search* button, which is located at the top of the web page, next to the drop-down list with the names of databases.

Moreover, the module of advanced searching also allows users to enter additional information in order to limit the number of query results. Constraints can be imposed on the range of publication dates or modification dates for records in the specified database. To use this feature, appropriate fields of the search form should be completed in the query builder (Figure 9, point 3).

Results of the constructed query are presented in the same way as results of the simple searches. The clipboard options and links are also available, and work in the same way for the results of queries made using the advanced search form.

### Creating macros

**Figure 10:**
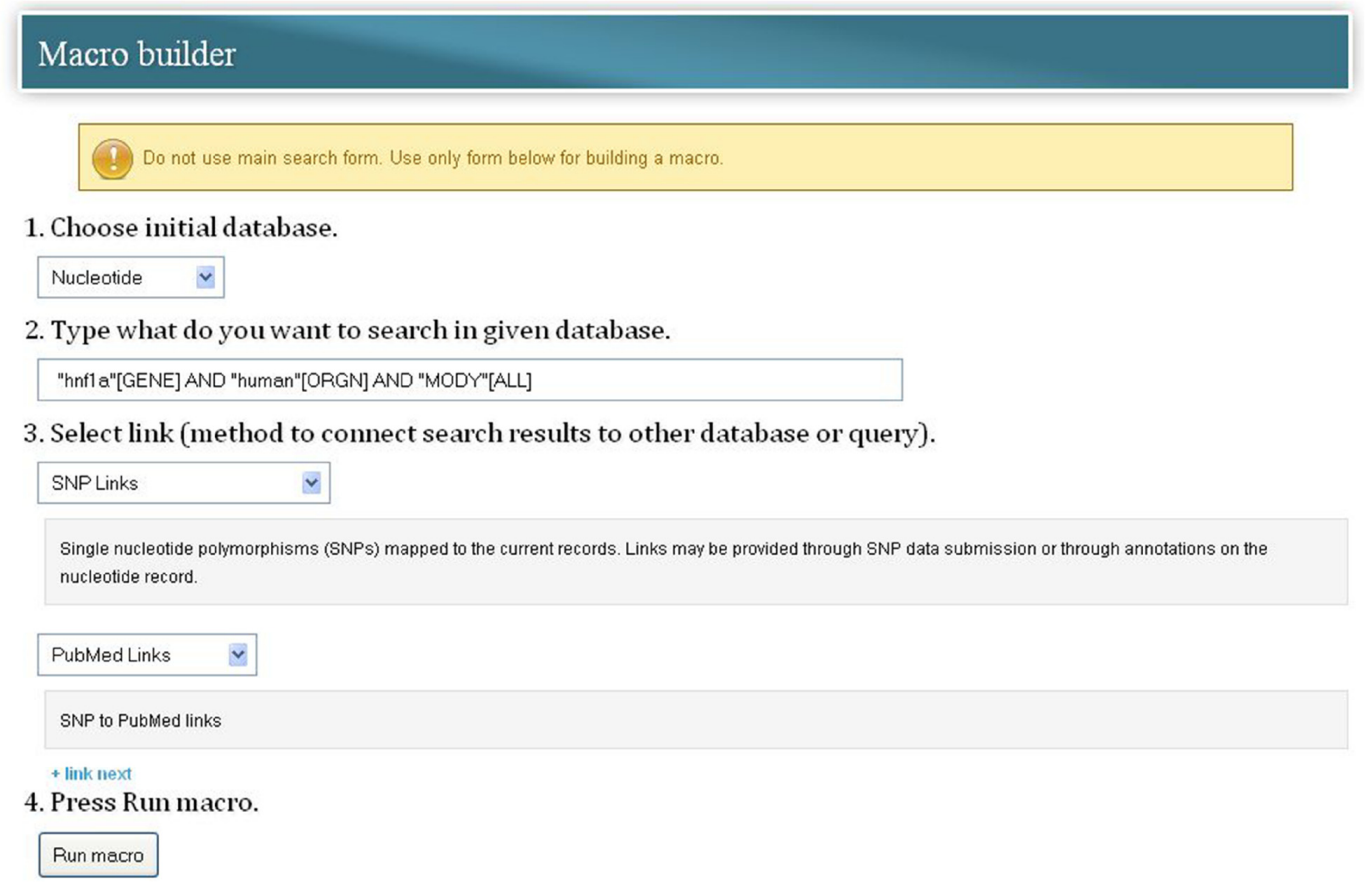
**Creating macros in *search GenBank *web portal.** In this example, a user wants to obtain a list of scientific articles that are related to all single nucleotide polymorphisms (SNPs) mapped to nucleotide sequences corresponding to human *HNF1A* gene. A user chooses an initial database (*Nucleotide*, point 1) and enters a query (e.g. *“hnf1a”[GENE] AND “human”[ORGN] AND “MODY”[ALL]*) that will be executed against this database (point 2). Then, he/she chooses additional links that allow traversing to other databases (*SNP* and *PubMed*). In order to add another link he/she can use the *+ link next* hyperlink. When the macro is completed, it can be executed by pressing the *Run macro* button.

Macros allow users to discover more biological information about particular data by following the links defined between databases. They automate the search for records which are related to records from another or from the same database. For example, starting with nucleotide sequences of the *HNF1A* gene returned from the GenBank, we can automatically follow all single nucleotide polymorphisms (SNPs) mapped to the current records and then, all scientific articles from *PubMed* related to these SNPs. Such macros can be created by using the appropriate form available in the *search GenBank* service. This form is presented in Figure 10.

In order to construct a macro, it is necessary to specify the name of the initial database in which to search records that match the entered query. Then, from a shared list, the user chooses additional links that allow traversing to other databases or the same database, when looking for neighbors. After the macro is completed, it can be executed and saved in the dictionary of macros, provided that you are logged in as a registered user.

It should be noted that macros do not show records from the initial and intermediate databases and do not require reviewing and selecting any records. Users obtain a list of records from the last of the linked databases. Theoretically, the number of links entered into the macro is infinite, but we should take into account the fact that not all of the records in the NCBI databases have annotations linking related items in other databases. However, over time, the number of links between the NCBI databases will grow. Therefore, we can be optimistic that, in the future, macros will prove to be a great alternative to the laborious, manual exploration between databases.

Other examples of macros are shown in Table 3.

**Table 3:** Sample macros

**Problem:***Find all genes for amino acid sequences, corresponding to a protein called: topoisomerase*		
**Query**	**Link**	
topoisomerase[protein name]	Gene Links	
**Database**		
Protein	**Found:** 20 records	
**Problem:***Find nucleotide sequences for mouse, and then all articles available in the PubMed that are related with the nucleotide sequences*		
**Query**	**Link**	
mouse	PubMed Links	
**Database**		
Nucleotide	**Found:** 785 records	
**Problem:***Find all possible records from the PopSet database corresponding to the breast cancer, then search the related nucleotide sequences. Bind the found nucleotide sequences with protein sequences.*		
**Query**	**Link**	**Link**
Breast cancer	Nucleotide Links	Protein Links
**Database**		
PopSet		**Found:** 1081 records

### Discussion

Construction of the *search GenBank* and possibilities provided by the system confirm that the role of Web services in the integration and exploration of various biological resources is important. It also shows how the coordination of the complex workflows over Web services can be achieved by orchestration and choreography.

Depending on the exploration mode, the *search GenBank* serves as an interactive orchestrator or choreographer executing various exploration paths. Comparing the *search GenBank* system to other tools that orchestrate Web services, like Taverna and SADI, and also commercial MapForce and SQL Server Integration Services, we can say that these tools have a universal purpose and provide the possibility to explore a broader range of resources and Web services. SADI gives even more by adding semantics to the discovery of distributed data resources. However, the above mentioned tools have different target users. These can be, for example, specialists in bioinformatics and data flow architects, whereas *search GenBank* is dedicated, e.g. to biochemists, biologists and medical doctors. Users of *search GenBank* do not have to take care of the implementation details, Web service interfaces, domain specific query languages, and how to connect inputs and outputs of particular tools. These details are hidden under the *search GenBank* web GUI that our system provides. In *search GenBank,* exploration paths can be constructed dynamically and users are able to traverse between various data sources whilst still having an open door to take another step. On the other hand, while working with macros, users construct predefined traverse paths that are similar to workflows in the Taverna and SQL Server Integration Services, although with limited possibilities, but in a simpler manner through the friendly graphical user interface that is appropriate for them.

**Figure 11:**
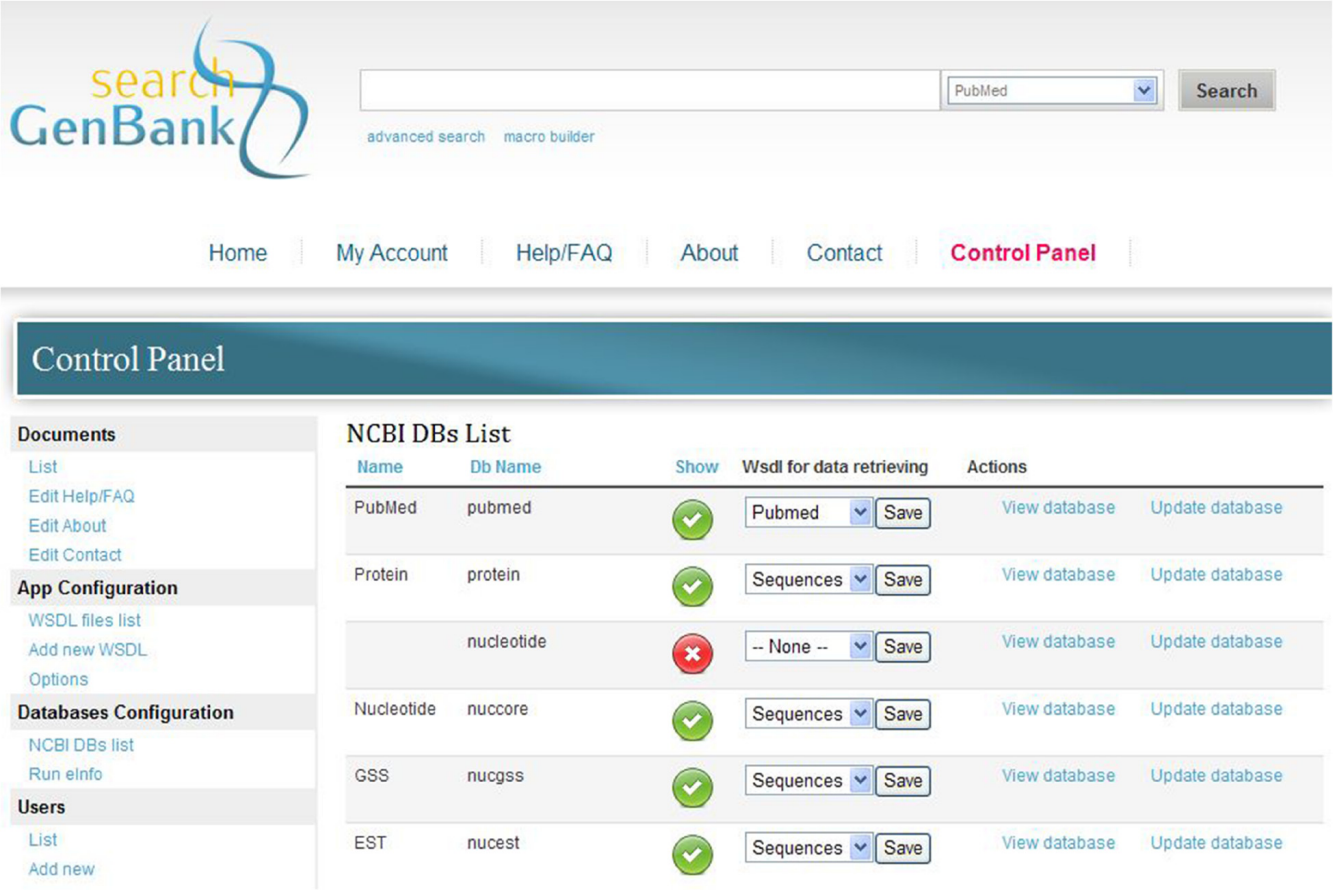
***search GenBank *system administration using Control Panel.** Control Panel allows administration of the *search GenBank* system, i.e. editing its content, adding new data resources and registering new WSDL files describing Web services, configuring databases, administering security (see left-side menu). The central panel displays the list of NCBI database resources with dropdown lists providing the possibility to map a database to a particular, registered WSDL file. WSDL registration is possible though the *Add new WSDL* hyperlink in the *App Configuration* section of the left-side menu.

*search GenBank* is still extendable. Similarly to other tools, *search GenBank* also allows the addition of new WSDL files describing new Web services. However, this is only possible for administrators of the system through the special web form that is presented in Figure 11.

In the future, we plan a further development of the system by extending its capabilities in accessing biomedical resources available world-wide. We want *search GenBank* to provide an integrated access to distributed tools and biological data from various scientific repositories, hiding the implementation details of a particular set of functionality under a friendly graphical interface.

## Conclusions

*search GenBank* provides an internet portal that allows simple and advanced data searching in GenBank and other databases which are maintained by the National Center for Biotechnology Information. Furthermore, the possibility of creating macros allows for cross-database exploration of related data. This is a unique feature of the *search GenBank* system. Currently, the strength of this solution may not be fully utilized, due to the fact that the current relationships between records of different databases are not very complex. However, we believe that the potential of the idea that lies in the automation of finding useful information in biomedical databases will grow with the increasing density of relationships between data stored in particular databases.

The *search GenBank* system has been designed for people involved in the analysis of biological data, including biochemists, molecular biologists, medical doctors, staff of genetic laboratories and molecular pathologists. Registered and logged in users of the system can save queries and macros in the special dictionaries, so that, in the future, when conducting similar studies, they can reuse them.

*search GenBank* complements the capabilities of the NCBI Entrez portal. It concentrates largely on genetic data, based on the assumption that genetic data are currently the most frequently used data in life sciences. However, it also allows data searching and data exploration in other NCBI databases.

## Availability and requirements

**Project name:** search GenBank

**Project home page:**http://zti.polsl.pl/dmrozek/science/sgb/sgb.htm

**Operating systems:** Platform independent

**Programming language:** PHP

**Other requirements:** Apache 2.2.10 or higher, MySQL 5.1.30 or higher, PHP 5.2.6 or higher with the following extensions: com_dotnet, ctype, session, filter, ftp, hash, iconv, json, odbc, pcre, date, libxml, standard, tokenizer, zlib, SimpleXML, dom, SPL, wddx, xml, xmlreader, xmlwriter, apache2handler, curl, gd, mbstring, mysql, mysqli, PDO, pdo_mysql, soap, SQLite

**License:** free for academics

**Any restrictions to use by non-academics:** licence needed

## Competing interests

The authors declare that they have no competing financial interests.

## Authors’ contributions

AS and DM were developers of the *search GenBank* system. DM was the manager and supervisor of the project. He defined functional requirements for the system and designed possible architectures. AS was a main software implementer. BMM was an advisor in designing and testing the system. BMM, DM and AS equally contributed to the paper. All authors have read and approved the manuscript.
